# The zebrafish anatomy and stage ontologies: representing the anatomy and development of *Danio rerio*

**DOI:** 10.1186/2041-1480-5-12

**Published:** 2014-02-25

**Authors:** Ceri E Van Slyke, Yvonne M Bradford, Monte Westerfield, Melissa A Haendel

**Affiliations:** 1 ZFIN, 5291 University of Oregon, Eugene, OR 97403-5291, USA; 2 Institute of Neuroscience, 1254 University of Oregon, Eugene, OR 97403-5291, USA; 3 Ontology Development Group, OHSU Library, 181 Southwest Sam Jackson Park Road, Portland, OR 97239-3079, USA

## Abstract

**Background:**

The Zebrafish Anatomy Ontology (ZFA) is an OBO Foundry ontology that is used in conjunction with the Zebrafish Stage Ontology (ZFS) to describe the gross and cellular anatomy and development of the zebrafish, *Danio rerio*, from single cell zygote to adult. The zebrafish model organism database (ZFIN) uses the ZFA and ZFS to annotate phenotype and gene expression data from the primary literature and from contributed data sets.

**Results:**

The ZFA models anatomy and development with a subclass hierarchy, a partonomy, and a developmental hierarchy and with relationships to the ZFS that define the stages during which each anatomical entity exists. The ZFA and ZFS are developed utilizing OBO Foundry principles to ensure orthogonality, accessibility, and interoperability. The ZFA has 2860 classes representing a diversity of anatomical structures from different anatomical systems and from different stages of development.

**Conclusions:**

The ZFA describes zebrafish anatomy and development semantically for the purposes of annotating gene expression and anatomical phenotypes. The ontology and the data have been used by other resources to perform cross-species queries of gene expression and phenotype data, providing insights into genetic relationships, morphological evolution, and models of human disease.

## Background

Zebrafish (*Danio rerio*) share many anatomical and physiological characteristics with other vertebrates, including humans, and have emerged as a premiere organism to study vertebrate development and genetics [1]. Zebrafish are amenable to genetic manipulation, and several techniques allow recovery of zebrafish mutations affecting developmental patterning, organogenesis, physiology, behaviour, and numerous other biological processes [2-4]. In addition to genetic screens, zebrafish are amenable to targeted gene knock-down utilizing morpholino antisense oligonucleotides (MOs) [5], TALENs [6], and CRISPRs [7]. Use of transgenic constructs in zebrafish has further expedited the study of gene function [8,9]. These various methods for altering gene expression and regulation have generated a plethora of data that enable modelling of disease states and that provide a greater understanding of gene function, development, and evolution. ZFIN, the zebrafish model organism database [10] manually curates these disparate data obtained from the literature or by direct data submission.

ZFIN serves as the central repository for zebrafish genetic, genomic, phenotypic, expression, and developmental data and provides a community web based resource to enable access to this highly integrated data [11,12]. To support annotation of gene expression patterns and phenotype information in wild types and fish with altered gene function, ZFIN has developed the Zebrafish Anatomy Ontology (ZFA) [13] and the Zebrafish Stage Ontology (ZFS) [14]. By using the ZFA and ZFS to annotate gene expression and phenotypic data, ZFIN is able to provide efficient querying and analysis across ZFIN data as well as cross-species inference [15]. ZFIN is actively involved in the zebrafish and ontology research communities to improve the ZFA through addition of classes, definitions, relations, and common design patterns and efforts towards interoperability with other ontologies. We report here on the design of the ZFA and ZFS, the current state of the ontologies, and ongoing efforts to maintain these ontologies for representing the knowledge of zebrafish and more broadly, vertebrate anatomy.

## Results

### Design considerations ZFA

**Figure 1:**
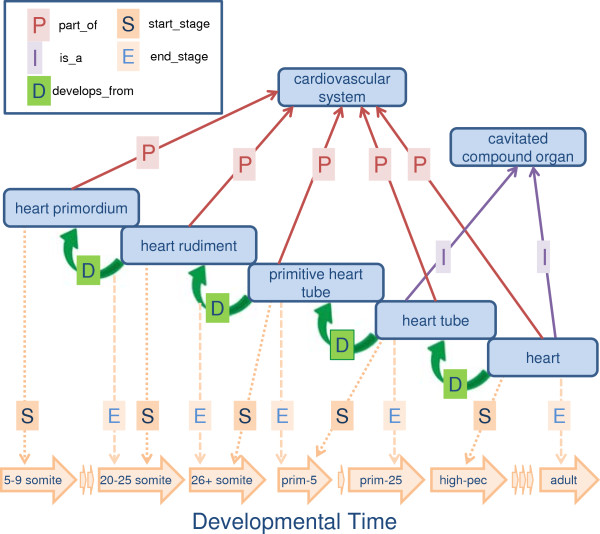
**Ontological representation of zebrafish heart development.** The ontological representation of heart development includes overlapping start and end stages where the start stage of a developing structure is the same as the end stage of the previous structure, and abutting, where a structure ends the stage before the start stage of the next structure. Overlapping and abutting start and end stage design considerations are illustrated: 1) start_stage and end_stage temporally overlap for ‘primitive heart tube’ (ZFA:0000149) end_stage ‘Pharyngula:Prim-5’ (ZFS:0000029) and ‘heart tube’ (ZFA:0000360) start_stage ‘Pharyngula:Prim-5’ (ZFS:0000029); 2) end and start stages abut for ‘heart tube’ (ZFA:0000360) end_stage ‘Pharyngula:Prim-25’ (ZFS:0000031) and ‘heart’ (ZFA:0000114) start_stage ‘Pharyngula:High-pec’ (ZFS:0000032). Note: several is_a parents omitted from the figure for clarity. Stage arrows indicate stage order not length.

The ZFA ontology includes a representation of the anatomy of *Danio rerio* at all stages of life - from a single-cell zygote to an adult. The main features of the ZFA, in addition to its largely structure-based subclass hierarchy, are its partonomy (using the part_of relation) and developmental hierarchy (using the develops_from relation). Each anatomical class in ZFA is defined using these relationships to other classes in ZFA as well as to stage classes in ZFS. The relations used in the ZFA and ZFS ontologies are listed in Table 1, and include start_stage and end_stage. The start_stage utilized is equivalent to Relation Ontology (RO) [16] ‘starts_during’ and end_stage is equivalent to RO ‘ends_during’. In this way, each anatomical entity can be defined in terms of what it is a type of, what it is a part of, what it develops from, and during which stages it exists. Figure 1 shows an example of how the ZFA describes the development of the heart and illustrates the relationships used to describe the partonomy, developmental series, and relationships between anatomical entities and stages.

**Table 1:** Examples of relationships used in the ZFA and ZFS

**Property**	**Explanation**	**Examples**
is_a (subclass in OWL)	Subtypes a class by its intrinsic nature.	The heart is_a cavitated compound organ.
part_of (BFO:0000050)	Describes what structure or system the class is a part of.	The heart is part_of the cardiovascular system.
develops_from (RO:0002202)	Describes a class by its progenitors.	The heart develops_from heart tube.
start_stage (RO:0002091)	Describes a class that is observed to begin during a particular stage.	The heart has a start_stage of Pharyngula:High-pec
end_stage (RO:0002093)	Describes a class that is observed to end during a particular stage.	The heart has an end_stage of Adult
immediately_preceded_by (RO:0002087)	Describes the order in which process classes occur in time.	Pharyngula:High-pec is immediately_preceded_by Pharyngula:Prim-25

The ZFA was developed based on the original zebrafish anatomical dictionary, which was a loosely structured partonomy. The anatomical dictionary was developed to: 1) computationally disseminate gene expression and phenotypic data; 2) define the anatomical structures of the zebrafish to establish an ontological framework that could be used by all zebrafish researchers to describe and contribute data; 3) provide an interoperable anatomical description of zebrafish to effectively map relationships between analogous structures across species [17]. The biologists involved in conceptualizing the ZFA used the anatomical dictionary as a framework and structured the ZFA according to the original version of the Common Anatomy Reference Ontology (CARO) [18] at its upper levels of organization, making the ZFA interoperable with other ontologies built using CARO as a framework. This is in contrast to alternative approaches taken by the Mouse Gross Anatomy and Development Ontology (EMAP) [19,20], or the Drosophila gross anatomy (FBbt) [21], where a partonomy is represented for each developmental, or life, stage. In addition to the standard CARO classes, the ZFA includes an additional high level term ‘embryonic structure’ (ZFA:0001105)^a^, to organize embryonic tissues described by fate mapping or gene expression in the early embryo. This class is especially useful for organizing presumptive anatomical structures or areas described as anlagen, primordia, or undifferentiated buds. These developmental classes are difficult to classify structurally, without use of more complex class expressions, thus it makes more sense for the user to organize these classes based on ontogeny. Structural representation of such entities was originally described by the CARO developers [18], but as was noted, requires enhancement based on ontogeny.

ZFA classes have human-readable text definitions that usually are structured in the genus-differentia format as recommended in Smith et al., 2007 [22] and codified in the 2008 OBO Foundry principles [23]. Class definitions also include further biological description to aid in the identification and understanding of zebrafish anatomy structures by the user or annotator. The ZFA does not have logical (computable) definitions at this time, though these are targeted for future development. In the meantime, many computable definitions for ZFA classes can be found in the uberon-zfa file [24].

In an effort to include cell terms in the ZFA needed to support partonomy-based queries, we incorporated the appropriate leaf nodes of the Cell Ontology (CL) [25]. Reusing CL classes instead of making new zebrafish cell classes allows the ZFA to be more interoperable with the other OBO foundry ontologies [26]. To accommodate proper reasoning using these species-independent classes, the file header includes the line “treat-xrefs-as-genus-differentia: CL part_of NCBITaxon:7955” that informs users and applications that the terms with CL cross-references are zebrafish-specific subclasses of cells in the CL [27]. In this way, the CL terms needed for cross-granularity partonomy queries were added to the ZFA such that each term was incorporated with a ZFA identifier and a cross reference to the CL term ID. Currently there are 426 cell terms with cross references to the CL and 109 of those terms have a part_of relationship with a zebrafish structure. The ZFA editors work closely with the CL editors to ensure classes added to the CL are appropriate for use in the ZFA as well as for other Teleosts and Tetrapods. For cells that are uniquely found or named in zebrafish such as ‘MiP motor neuron’ (ZFA:0005179), classes are added only to the ZFA and not to the CL. The zebrafish specific classes are positioned as subclasses within the imported CL class hierarchy.

During the early stages of ZFA development, it was difficult to gain zebrafish-specific community input. To overcome this, developers convened zebrafish specific working groups focusing on blood [28], skeletal system [29], digestive system [30], and nervous system [31]. This strategy provided community input at critical junctions and resolved long-standing discrepancies and conflicting views about various aspects of zebrafish development. To acknowledge the invaluable contributions made by these working groups, participants were given a virtual publication at ZFIN, allowing concepts developed by the working group to be referenced in the ZFA while publicly acknowledging their contributions. Such grey-literature publications can be referenced directly in the ontology, similar to the Uberon design document system [32].

The ZFA has a complete subclass hierarchy, where 81% of the 2860 terms have text definitions, 1733 terms have part_of relationships, and there are 459 develops_from relationships. The maximum tree depth of the ontology is 21 nodes. The ZFA has added 900 new terms since May of 2007, a rate of 180 a year, and added or updated 1120 definitions, a rate of 224 a year. We identify anatomy terms for inclusion in the ZFA from zebrafish literature, zebrafish specific working groups, workshops focusing on a specific organ system such as the vertebrate skeletal system [33], or through the project’s SourceForge tracker [34].

Before a new version of the ZFA is released, we run a series of quality control checks on the ontology to ensure stage consistency in the partonomy, subclass hierarchy, and the developmental hierarchy. Checks performed on the subsumption and partonomies ensure that child classes have stages that are the same as the parent class or that the stages fall within the range of the parent class. The stage relation requirements for the developmental hierarchy are slightly different. A structure that has a develops_from relationship with another structure must have a start stage that is either the same as (overlapping), or one stage later (abutting) than the structure it develops from see Figure 1. After the ontology has passed the quality control checks and before it is released, we check the expression annotations to ensure that the stages to which the terms are annotated do not conflict with the stages delineated in the ZFA. Phenotype annotations do not need to meet these stage constraints because phenotypes often involve the delayed development of a structure.

### Content considerations ZFA

Zebrafish is a research organism undergoing active description of anatomical structures and development. Therefore, many structures known to be present in other model systems have yet to be described in zebrafish or the state of knowledge about the ontogeny is questionable. This means that certain nodes of the ontology are incomplete relative to analogous or homologous structures in other species. For example, a term stomach had been included within the original anatomical dictionary, but later research that defined stomach based on the constituent cell types suggested that in fact zebrafish do not have a stomach. This was confirmed by consultation with a researcher who sent histological sections, and the class was removed. Similarly, the zebrafish do not have a pons by most definitions. Conversely, while the ventricular epicardium had been described in zebrafish in electron micrographs [35], the atrial epicardium had not. More recently, transgenic zebrafish lines that specifically label the atrial epicardium were constructed in 2010 [36] and the class ‘atrial epicardium’ (ZFA:0005774) was recently added to the ontology.

When the ontogeny of a structure isn’t agreed upon, a class representing the structure is not added to the ontology until researchers come to a consensus, an example of such a structure is dermomyotome. As late as 2001, it was stated “Somewhat surprisingly, the epithelial structure of the dermomyotome itself, which in amniotes gives rise to both the myotome and the dermatome, does not seem to have a direct functional ortholog in zebrafish” [37]. In 2006, the dermomyotome was described [38], but full characterization is still underway [39]. Accordingly, dermomyotome has been added to the ontology but a more complete representation awaits further research. A similar example can be seen in the case of lymph vessels. Most organisms have lymph vessels, but in zebrafish their existence has been controversial. Very early papers identified structures as lymph vessels, but later researchers could not corroborate this assertion. Lymph vessels were finally described in 2006, however no lymph nodes have been identified [40,41]. In light of the controversy invoked by the very suggestion that certain structures exist in zebrafish, it is difficult to decide what standard of evidence is required to add a class to the ontology: based on homology to other organisms, at the first mention in the literature, or after full characterization? Since ZFA classes are required for literature annotation, the ZFA editors add classes to the ontology as they are described in the literature, through discussion and coordination within the larger anatomy ontology community, and through individual anatomy term requests.

### Design considerations ZFS

To support representation of developmental time in the ZFA, we developed the ZFS concurrently. The ZFS contains a representation of the community standard developmental staging series, based on the development of landmark anatomical structures [42,43]. It has a shallow subclass hierarchy of stages modelled as an ordered set of processes organized by the immediately_preceded_by relationship between stages, and part_of relationships to “super-stages” (e.g. ‘embryonic stage’). The maximum tree depth of the ontology is 3 nodes. The content of the ontology has been stable since its release. Classes in the ZFA are related to the ZFS leaf nodes and not the super-stages, which are used solely for querying purposes. Every anatomical entity in the ZFA has a start_stage and end_stage relationship to ZFS stages. For ZFA terms where the start or end stage has not been determined, the ZFS class ‘unknown’ (ZFS:0000000) that starts at 1 cell and goes through adult is used (encompassing the entire developmental series). All ZFA classes must have either a known start stage or a known end stage, such that there is no anatomical entity with the relationship start_stage ‘unknown’ (ZFS:0000000) and end_stage ‘unknown’ (ZFS:0000000). For annotating anatomy terms that have a complex developmental time line, we use terms that have abutting or overlapping start_stage and end_stage to model the developmental progression of the tissue or entity.

### Anterior/posterior modeling

**Figure 2:**
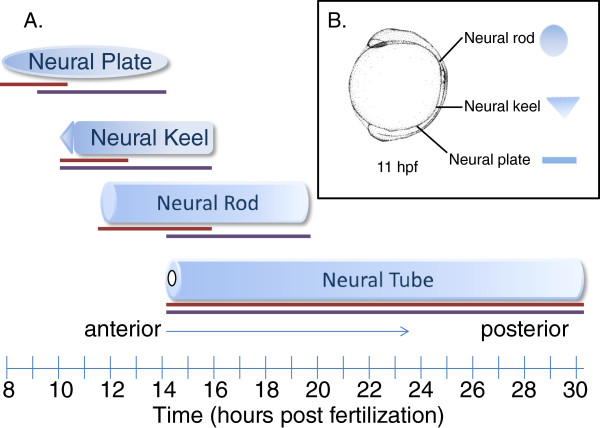
**Representing anterior-posterior (rostral-caudal) development of midline. A**. Developmental progression of the neural plate, neural keel, neural rod, and neural tube. The brick line denotes the time period when the anterior portion of the above structure is developing, the purple line represents the same for the posterior portion of the structure. The anterior and posterior part_of children of the structure correspond to the colored lines. Small differences in start times between the anterior and posterior regions are not describable due to the length of the developmental stage. **B**. Schematic cross section of neural tissue at different anterior-posterior positions at the same stage, representing the different morphologies of structures. The neural plate extends from the anterior end of the organism (the head) to the posterior end (the tail). The neural plate is a flat expanse of cells; it develops into a group of cells with a triangular cross section, the neural keel. The neural keel changes from a triangular cross section to a round cross section as it forms the neural rod. Finally, the neural rod cavitates to form a hole down the center, giving it a donut shaped cross-section. The anterior end develops the characteristics of the next structure, while the posterior end is a few morphological phases slower, e.g. the posterior of the organism is still neural plate when the anterior has started forming a rod morphology.

One of the challenges of representing bilaterian development in an ontology is that structures form at different times along the primary axes. For example, the zebrafish neural rod undergoes cavitation along the anterior-posterior axis to form the neural tube [44]. This process proceeds from the anterior end of the embryo, completing formation of the neural tube before the posterior end of the neural rod has formed. Our solution was to model the varying morphologies of neural tissue along the anterior posterior axis to represent the progression of the entire structure over time. Each class has spatially localized parts that have overlapping or abutting stages as appropriate, see Figure 2. The anterior/posterior division of the axial neural structures follows the developmental progression of the neural tube, with the anterior portions developing into the brain and the posterior region developing into the spinal cord. A similar axial developmental pattern is followed during somite formation. In the case of somites, however, bona-fide boundaries form so the development of each somite is represented individually.

### Using the ZFA and ZFS for data annotation

ZFIN curators use the ZFA and ZFS to annotate expression and phenotype data as described in the primary literature. Gene expression is annotated for individual genes in a particular structure at a specific stage, along with the assay used to detect expression, for wild-type fish and fish with altered gene function. Lack of gene expression is recorded if noted by authors. The ZFA is also used to annotate structures in reference anatomical histology slides for the Zebrafish Atlas project [45] and may be used for annotation by other atlas projects giving the community a common set of terminology.

ZFIN utilizes the ZFA in combination with other biomedical ontologies like the Spatial Ontology (BSPO; this issue) [46] and Gene Ontology (GO) [47,48] to annotate gene expression in more specific structures using a post-composition approach [49]. This approach allows more expressive terms to be created on the fly without having to enumerate all possible classes *a priori*, prevents very granular proliferation of terms, and maintains interoperability with other ontologies and data sources. ZFIN curators utilize post-composition in several ways: 1) create an anatomy term that describes the anatomical structure by post composing a ZFA class with another ZFA class, or GO Cellular Component Ontology(GO-CC) class; 2) create a class that imparts spatial location by post-composing the ZFA term with a class from the BSPO (see Table 2). Post-composed classes are logically consistent with the overall graph structure of the ontologies, since only the part_of relation is used to relate these classes. Closure over these post-composed classes is performed within the ZFIN database to ensure consistency.

**Table 2:** Examples of Post-composed classes

**Type of Post-composition**	**Classes**	**Manchester Syntax**
ZFA + ZFA	‘motor neuron’ + ‘spinal cord’ (ZFA:0009052) (ZFA:0000075)	‘motor neuron’ that part_of some ‘spinal cord’
BSPO + ZFA	‘dorsal region’ + ‘spinal cord’	‘dorsal region’ that part_of some ‘spinal cord’
	(BSPO:0000079) (ZFA:0000075)	
GO-CC + ZFA	‘axon’ + ‘motor neuron’	‘axon’ that part_of some ‘motor neuron’
	(GO:0030424) (ZFA:0009052)	
MPATH + ZFA	‘Melanoma’ + ‘head’	‘Melanoma’ that part_of some ‘head’
	(MPATH:359) (ZFA:0001114)	

**Figure 3:**
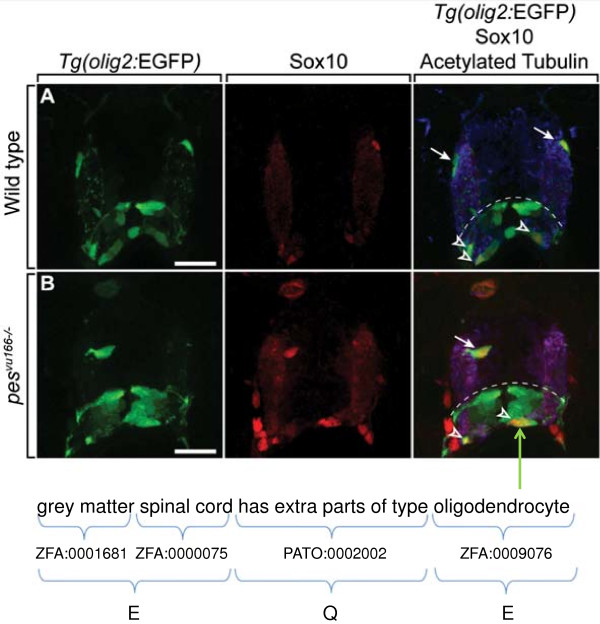
**Representing phenotype annotations using postcomposition and EQE syntax.** In the image from Simmons et al., 2012 [53], *pes*^*vu166/vu166*^*Tg(olig2:EGFP)vu12* fish **(B)** have an increased number of oligodendrocytes (green) in the grey matter of the spinal cord compared to WT **(A)**. This is annotated by the EQE statement “grey matter part_of spinal cord has extra parts of type oligodendrocyte”. In this annotation ‘spinal cord’ (ZFA:0000075) is post-composed with ‘grey matter’ (ZFA:0001681) to indicate that the phenotype is visible in the grey matter of the spinal cord; the PATO term used is relational which requires the use of a second entity term, in this case ‘oligodendrocyte’ (ZFA:0009076).

ZFIN curators also annotate phenotypes from the published literature or direct submission for mutant and transgenic fish at a particular stage, along with data from fish analysed by gene knockdown technologies. Phenotype data are annotated using the Entity-Quality (EQ) or Entity-Quality-Entity (EQE) convention using post-composition [15]. Entity terms can be continuants or occurrents, whereas Quality terms come from the Phenotypic Quality Ontology (PATO) [50,51]. Continuant entities are chosen from the ZFA and can be post-composed with GO-CC, BSPO, or Mouse Pathology Ontology (MPATH) [52] neoplasm terms to further specify cellular, subcellular, spatial location, or disease state of the entity. Occurrent entities are selected from the GO Biological Process Ontology (GO-BP) or the GO Molecular Function Ontology (GO-MF). We use the EQE syntax to describe a phenotype that relates two entities, which allows use of an additional entity with the relational qualities found in PATO. For example, we used EQE syntax to annotate the phenotype observed in the *pes*^*vu166/vu166*^*; Tg(olig2:EGFP)vu12* mutant reported in Simmons *et al*., 2012 [53] (Figure 3). We capture normal phenotype data, i.e. normal phenotypes observed in mutants, if the authors describe them as notable. ZFIN accepts community data submission if the data are submitted using Phenote [54], an annotation tool that facilitates EQ and EQE annotations by providing a list of entities from appropriate ontologies and qualities from PATO.

### Search using ontology terms

**Figure 4:**
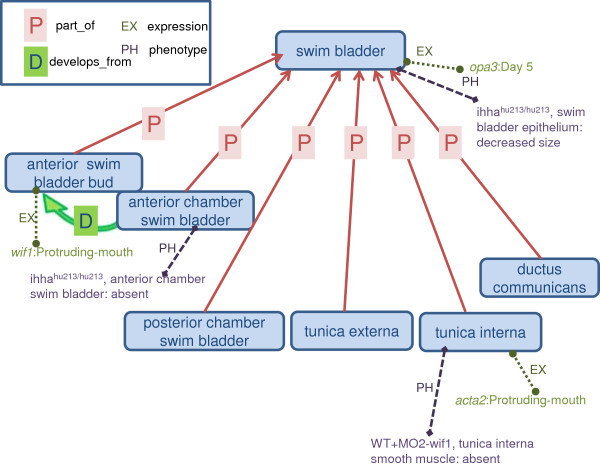
**Illustration of query expansion used in gene expression and phenotype searches.** When a user searches for a particular term, such as ‘swim bladder’, any annotations to classes with a part_of relationship to ‘swim bladder’ are returned. For example, searching for expression in ‘swim bladder’ would return expression annotated to ‘swim bladder’, *opa3* at Day 5, as well as *wif1* expression in the ‘anterior swim bladder bud’ and *acta2* expression in the ‘tunica interna’ at ‘Protruding mouth’ stage. Searching for expression in ‘anterior swim bladder bud’ would return expression of *wif1* only at ‘Protruding-mouth’. The same logic is used for returning phenotype data. Phenotypes affecting ‘swim bladder’ occur in *ihha*^*hu213/hu213*^ fish that have ‘swim bladder epithelium’ with decreased size and the ‘anterior chamber swim bladder’ is absent, as well as for wild-type fish treated with MO2-wif1 where the (‘smooth muscle’ part_of ‘tunica interna’) is absent.

In addition to standard autocomplete functionality in ZFIN, we leverage some of the ontology relations to support queries of gene expression and phenotype data. Users instinctively expect interfaces to return classes in which the class is an ancestor in subclass and part_of hierarchies. ZFIN utilizes query expansion that leverages ontologies to support this requirement (Figure 4). Post-composed terms using a primary ZFA class and secondary BSPO, GO_CC, ZFA, or CL class are children of the primary ZFA term and are treated as such by the search algorithms.

### Using the ZFA as a collaborative framework

The ZFA has an interoperable framework that several ontologies have adopted either through direct cloning or active consultation of ZFA architecture and editors. The first species specific ontology to utilize the ZFA framework was the Medaka AO, where ZFA ontology editors exchanged either Filemaker templates or plain text files with the Medaka AO developer who then incorporated desired changes. The Medaka AO was developed to annotate gene expression patterns, has continued to grow and has been supplanted by the Medaka fish anatomy and development ontology (MFO) [55]. The Xenopus Anatomical Ontology (XAO) [56], is another example of a species specific ontology that began under the guidance of ZFA editors, closely replicating the architecture of the ZFA as it was developed to annotate gene expression and phenotype data by Xenbase curators [57]. The Teleost Anatomy Ontology (TAO) [58] was directly cloned from the ZFA to create a general ontology to represent the diverse anatomy of fish found in the taxon Teleosti. The TAO was cloned from the ZFA utilizing a Perl script that transformed a copy of the ZFA by changing the identifier prefix of each ZFA term to a TAO identifier and adding cross references for each TAO term to a ZFA term [59]. The TAO was then generalized to be applicable to all Teleosti by removing ZFA adult anatomy classes that do not generalize across teleosts and relationships to ZFS. ZFA terms are logically subtypes of TAO terms. The first iteration of the TAO was used to annotate wild type phenotype statements captured as part of the Phenoscape project [60]. The TAO has subsequently been subsumed by UBERON, as described in this issue [61].

## Discussion

The ZFA and ZFS ontologies were designed by ZFIN to describe the anatomy and development of zebrafish according to OBO Foundry principles. The ZFA was initially developed to define zebrafish anatomical structures within an ontological framework that could be used by researchers to computationally disseminate gene expression and phenotypic data and provide an interoperable means of mapping relationships across species. The ZFA was originally based on the EMAP ontology structure, such that each stage had its own partonomy. To support our user interface query expansion, we converted the ontology to the representation described above, e.g. where each anatomical structure is related to others via subclass, part_of, and develops_from relationships, and is tied to stages via start and end relations to ZFS. This has proved an effective strategy not only for use in our user interface, but also for efficiency in data curation. Other anatomy ontologies have since followed similar design patterns (TAO, MAO, XAO, see above). In addition, the ZFA facilitated the creation of the multi-species ontology, Uberon. Uberon subsumes species-specific ontologies (including zebrafish, mouse, human, etc.) and thereby provides inference across taxa, enhancing the ability of users and applications to query expression and phenotypic data across species. Further, many additional fish-specific classes have been integrated into Uberon from the ZFA via the recent TAO integration and via regular quality assurance checks (see Uberon paper, this issue).

The ZFA continues to be developed as an interoperable and orthogonal ontology in the OBO foundry. In the future, ZFA will move to OWL and will leverage more sophisticated class axioms to describe anatomical entities, using classes from Uberon, CL, GO, and relations from the relations ontology using the MIREOT strategy [62]. This may include relations such as: has_muscle_insertion and has_muscle_origin to describe how muscles attach to bones, fasciculates_with (RO:0002101) and synapsed_by (RO:0002103) to describe the relations neurons have with other structures, capable_of (RO:0002215) and has_function_in (RO:0002216) to describe how structures participate in biological processes, and connects (RO:0002103) to describe interactions among structures. By including these relationships, more complete logical definitions can be constructed to support interoperability and more complex queries.

Active content development is focused on coordinating design patterns with members of the Uberon, MGI, and Phenoscape teams. For example, we are working on how best to represent anatomical boundaries that are the site of gene expression in anatomy ontologies (meaning they are not immaterial), bringing the skeletal system into compliance with the Vertebrate Skeletal Anatomy Ontology design patterns (VSAO [63], also recently integrated into Uberon), modeling zebrafish muscles, and defining all the undefined classes in the ontology.

The ZFA developers continue to seek community input on the design of the ontology by communicating with other species-specific (MA, FMA, EMAP) and species-neutral ontologies (GO, CL, Uberon [64]) through, OBO LISTSERVE and Phenotype RCN consortium [65] discussions that facilitate working through the larger issues that surround modeling of complex systems, such as neural crest development [66]. The design patterns agreed upon by the community can be realized across species-specific ontologies that utilize standardized anatomical classes, such as CARO, and that are tested and realized in Uberon.

The practical outcome of designing and using interoperable ontologies is that data annotated using the ZFA, and other interoperable ontologies, are configured such that it is simple for applications to use the data in cross-species comparisons. For example gene expression data that are annotated using the ZFA, and other biomedical ontologies, are utilized for cross-species gene expression comparisons at Bgee [67,68]. In addition to gene expression data, ZFIN produces phenotypic data that are utilized in cross-species queries. Phenotypic data curated by ZFIN, along with other Model Organism Database (MOD) phenotypic data, are incorporated into the Phenoscape Knowledge Base [69] where they can be queried to understand how genetic changes are linked to evolutionary changes [60]. Phenotypic data generated at ZFIN are also used computationally, in conjunction with mouse and human data, to understand human gene function and how genes are involved in disease processes [70-72]. ZFIN phenotype data have also been used to find the contribution of individual genes to human disease states caused by copy number variation [73] and in exome analysis [74]. The phenotype data generated by ZFIN, along with other MODs, are consumed by the LAMHDI/Monarch Initiative [75,76], which provides a web-based resource for biomedical researchers to access data about animal models of human disease.

The ZFA was built as an orthogonal, interoperable ontology and was designed using OBO Foundry principles. The ZFA meets all the goals of the original zebrafish anatomical dictionary.

## Conclusions

The ZFA and ZFS were developed by ZFIN to computationally describe zebrafish ontogeny and facilitate gene expression and phenotype annotation from the primary literature. In addition to its use in data annotation at ZFIN, the ZFA has been used as a seed ontology for several ontologies and has been successfully used by other resources for gene expression and phenotype comparisons. By development of the ontology and annotation of data using the ZFA and ZFS, ZFIN continues to provide a web-based resource for the zebrafish and broader biomedical research communities to understand zebrafish development in the context of human disorders and evolution. The continued development and participation of ZFIN curators in the greater ontology community will provide ongoing expansion of the ZFA and coordinated development. of interoperable resources. As MODs continue to generate anatomically annotated data, it is imperative that species specific ontologies continue to be interoperably developed to facilitate the translational phenotype analyses that will further our understanding of the evolution of the genome and the structures and process it produces.

## Methods

The ZFA was first released in 2004. The first zebrafish anatomical dictionary, which seeded the ZFA, was proposed May 10–11, 1999 [17] and implementation of the anatomical dictionary began December 1999 [77]. The ZFA is freely available from the OBO Foundry web site and is developed in OBO Edit 2.1 (OE2.1) [78] with the format version 1.2. Every two months an updated version of the ZFA is made available from the OBO Foundry [79]. The SVN release is noted in the file header. For expedient changes needed by collaborators, a pre-release download can be made available as needed. The edit version of the ontology is housed internally in the ZFIN SVN repository.

The OBO format version is deposited at Google Code zebrafish-anatomical-ontology project [80] and mirrored on the OBO Foundry web page, and the OBO Foundry generates the OWL file found at the OBO Foundry site.

ZFA follows the OBO Foundry best practices for ID generation and use. Each editor is assigned a stage range for term creation. IDs are never reused. If a term needs to be removed from the ontology, it is obsoleted and a consider or replaced_by tag is added if applicable. Classes that are found to be essentially the same are merged and the resulting class carries both IDs. Current ZFA ontology development practices dictate that all terms added to the ontology must be defined when they are added. If a definition needs to be changed rather than expanded, the class is obsoleted and a new class is created. Class names can change to reflect preferred community usage and exact synonyms are used to reflect other names for the structure. Class names in the ZFA are all singular. The ZFA uses an additional synonym type, ‘PLURAL’, to explicitly include the plural of the term name as a synonym. This capability is especially important for words with Greek or Latin roots where the plural is not created by appending ‘s’ to the term name.

The ontology version of the staging series, ZFS, was released in 2008. It is available from developmental-stage-ontologies at Google code [80]. The ZFS is freely available for download.

### Quality control checks and release

Prior to releasing a new ZFA version, quality control checks are run as described earlier. As a final check of the ZFA, the OBO Ontology Release Tool (OORT) [81] is used to reason over the ontology. Once the annotations and the new version of the ZFA have been checked, the official version is released to the public SVN repository. Users of the ontology are notified by email of the release of the new version and a summary of the major changes.

## Endnote

^a^Relations are denoted by the use of Courier font, while ontology classes are denoted by use of single quotes followed by term ID. Term IDs resolve to URIs by adding the ID in the format domain_number to the following URL, http://purl.obolibrary.org/obo/, e.g. http://purl.obolibrary.org/obo/ZFA_0001105.

## Abbreviations

BSPO: Spatial Ontology; CARO: Common Anatomy Reference Ontology; CL: Cell Ontology; CRISPR: Clustered Regularly Interspaced Short Palindromic Repeats; EMAP: Mouse Gross Anatomy and Development Ontology; EQ: Entity-Quality; EQE: Entity-Quality-Entity; FBbt: Drosophila Gross Anatomy; GO: Gene Ontology; GO-BP: GO Biological Process Ontology; GO-CC: GO Cellular Component Ontology; GO-MF: GO Molecular Function Ontology; MFO: Medaka Fish Anatomy and Development Ontology; MO: Morpholino Antisense Oligonucleotides; MOD: Model Organism Database; MPATH: Mouse Pathology Ontology; PATO: Phenotypic Quality Ontology; PATO: Phenotypic Quality Ontology; RO: Relation Ontology; TALEN: Transcription Activator-like Effector Nucleases; TAO: Teleost Anatomy Ontology; VSAO: Vertebrate Skeletal Anatomy Ontology; XAO: Xenopus Anatomy and Development Ontology; ZFA: Zebrafish Anatomy Ontology; ZFIN: Zebrafish Model Organism Database; ZFS: Zebrafish Stage Ontology.

## Competing interests

The authors declare that they have no competing interests.

## Authors’ contributions

MAH converted the zebrafish anatomical dictionary into the ZFA and maintained it, developed the ZFS, and contributed to the writing of the manuscript. YMB and CVS are responsible for current ontology development, and contributed to the writing of the manuscript. MW developed the original zebrafish anatomical dictionary, revised the manuscript, and supervised the development of the ZFA and ZFS. All authors read and approved the final manuscript.
